# The effect of fines on nonattendance in public hospital outpatient clinics: study protocol for a randomized controlled trial

**DOI:** 10.1186/s13063-016-1420-3

**Published:** 2016-06-13

**Authors:** Emely Ek Blæhr, Thomas Kristensen, Ulla Væggemose, Rikke Søgaard

**Affiliations:** DEFACTUM, Central Denmark Region, Olof Palmes Allé 15, Aarhus N, 8200 Denmark; Department of Public Health, Aarhus University, Bartholins Alle 2, Aarhus C, 8000 Denmark; Department of Clinical Medicine, Aarhus University, Palle Juul-Jensens Boulevard 82, Aarhus N, 8200 Denmark

**Keywords:** Fine, Nonattendance, Access to care, Outpatient clinic, Randomized controlled trial

## Abstract

**Background:**

Nonattendance at scheduled appointments in public hospitals presents a challenge for efficient resource use and may ultimately affect health outcomes due to longer waiting times. Seven percent of all scheduled outpatient appointments in the United Kingdom are estimated to be nonattended. Various reminder systems have been shown to moderately reduce nonattendance, although the effect of issuing fines for nonattendance has not yet been tested in a randomized context. However, such use of financial incentives could impact access to care differently across the different socioeconomic groups. The aim of this study is to assess the effect of fines on hospital outpatient nonattendance.

**Methods/design:**

A 1:1 randomized controlled trial of scheduled outpatient appointments was used, with follow-ups until the date of appointment. The setting is an orthopedic clinic at a regional hospital in Denmark. Appointments for users who are scheduled for diagnostics, treatment, surgery, or follow-ups were included from May 2015 to November 2015. Appointments assigned to the intervention arm include an attachment of the appointment letter explaining that a fine will be issued in the case of nonattendance without prior notice. Appointments assigned to the control arm follow usual practice (same system but no letter attachment). The primary outcome is the proportion of nonattendance. Secondary outcomes are proportions of cancellations, sociodemographics, and health-problem characteristics. Furthermore, the intervention costs and production value of nonattended appointments will be measured. An analysis of effect and cost-effectiveness will be conducted based on a 5 % significance level.

**Discussion:**

The study is initiated and funded by the Danish Regions, which have the responsibility for the Danish public healthcare sector. The results are expected to inform future decisions about the introduction of fines for nonattendance at public hospitals.

**Trial registration:**

Current Controlled Trials, ISRCTN61925912. Registered on 6 July 2015.

## Background

Recent hospital episode statistics from the United Kingdom show that approximately 20 % of scheduled outpatient appointments are nonattended [1]. While hospital and user cancellations each account for almost one third of the nonattended appointments, 7 % of nonattended appointments remain unaccounted for. Seven percent nonattendance is estimated to represent a cost to the healthcare system of around £790 million per year in the United Kingdom [2].

Outpatient nonattendance refers to the phenomenon of users who have an appointment but do not show up at the specified date, time, and location without giving notice. In addition to affecting the efficiency of production and thereby increasing the total costs of care, nonattendance might also delay access to care for users on waiting lists. Moreover, nonattendance has been suggested to have a detrimental effect on health outcomes [3, 4], an effect that is unevenly distributed across disease and socioeconomic groups [5, 6].

Stubbs et al. conducted a systematic literature review to assess the efficacy of various reminder interventions for reducing outpatient nonattendance such as telephone, mail, text/short message service, e-mail reminders, and open-access scheduling [7]. They found that almost all of the assessed interventions moderately reduced nonattendance. However, much of this evidence was based on observational studies, and most studies did not control for socioeconomic status and other relevant factors such as age, sex, waiting time, and health-problem characteristics. The recognition of a need for further high-quality studies has been confirmed by similar systematic reviews [8–10].

Another means for reducing outpatient nonattendance, for which there appears to be only vague empirical evidence, is to penalize nonattendance by a fine. According to neoclassical economic theory, user behavior can be influenced by financial incentives. The introduction of a fine for nonattendance will create a stronger incentive for users to appear at their scheduled appointments [11]. This has been demonstrated in two earlier observational studies where the introduction of fines led to a 14 % reduction and a 54 % reduction in nonattendance [12, 13]. These studies, however, only represent before-and-after scenarios, do not consider the likely influence of exogenous factors, and have limited sample sizes. Furthermore, the external validity is questionable due to the 20-year time lag.

In Denmark, the introduction of fines for nonattendance in hospital outpatient clinics has been debated for years [14]. In 2005, a collaborative agreement between the Danish Regions and the Danish Medical Association opened the opportunity for privately practicing specialists to issue nonattendance fines of Danish kroner (DKK) 250 for consultations and DKK 500 for surgical procedures (1 EUR = 7.45 DKK) [15]. Overall, the expected benefits were related to a better use of resources, whereas concerns were expressed about the detrimental effect on equal access for equal needs, as the fines would impact the most vulnerable users most strongly. Moreover, the use of fines was seen as a break with the fundamental value of free and equal access to healthcare and a potential threat to the patient-health professional relationship, which ideally should be independent of financial interests [14].

Although 10 years have passed since the regulatory context cleared the way for fines in the Danish healthcare system for privately practicing specialists, no systematic evidence on the resulting practice, efficacy, or effect appears to have been reported. Therefore, the Danish Regions have decided that the effect of using fines to moderate nonattendance at somatic, hospital-based, outpatient clinics in Denmark should be scientifically investigated [16]. This trial is a result of that decision and, in accordance, the aim of this trial is to investigate the effect of fines on nonattendance in a hospital-based outpatient clinic. The primary hypothesis is that the risk of fines will incentivize healthcare-service users to attend appointments more often than if no risk of fines is associated with nonattendance of a planned appointment. A secondary hypothesis is that the pattern of attendance will be affected by the risk of fines in terms of who attends (user characteristics) and what type of appointment is being attended (appointment characteristics). The rationale for the second hypothesis is to address the political focus on preserving equal access to healthcare and on variation in provider efficiency. Finally, a third hypothesis is that the use of fines in routine practice will be cost effective.

## Methods/design

A randomized controlled trial (RCT) will be conducted at a hospital-based orthopedic outpatient clinic in the Central Denmark Region. Randomization to an intervention arm (risk of fine) or to a control arm (no risk of fine) will be undertaken at the appointment level, starting in May 2015 and proceeding for 7 months. The trial conduct, analysis, and reporting will be in accordance with the Standard Protocol Items: Recommendations for Interventional Trials (SPIRIT) guidance 2013 [17] and the Consolidated Standards Of Reporting Trials (CONSORT) statement [18].

### Setting

The Department of Orthopedic Surgery at Viborg Regional Hospital consists of an outpatient clinic and a ward. The outpatient clinic has 18,500 scheduled appointments a year, of which approximately 19 % are cancelled and 5 % are nonattended (see Table 1). Medical secretaries manage the booking via an electronic booking system that automatically generates appointment letters that are sent to users. Apart from this system, a substantial number of appointments are scheduled without a sufficient time lag to allow for the dispersal of appointment letters (e.g., in cases of acute health problems) and thus are not considered in the present study.

**Table 1:** Historical number of cancelled (% of scheduled) and nonattended (% of noncancelled) appointments from April 2014 to March 2015 for the study setting

		Cancelled appointments			
	Scheduled appointments	By hospital	By user	Total	Nonattended appointments
April 2014	1,473	187 (13)	95 (6)	282 (19)	55 (5)
May 2014	1,467	223 (15)	105 (7)	328 (22)	64 (6)
June 2014	1,869	307 (16)	94 (5)	401 (21)	83 (6)
July 2014	1,082	175 (16)	66 (6)	241 (22)	50 (6)
August 2014	1,562	262 (17)	52 (3)	314 (20)	68 (5)
September 2014	1,661	270 (16)	151 (9)	421 (25)	62 (5)
October 2014	1,634	208 (13)	223 (14)	431 (26)	41 (3)
November 2014	1,744	205 (12)	89 (5)	294 (17)	71 (5)
December 2014	1,583	190 (12)	52 (3)	242 (15)	62 (5)
January 2015	1,511	166 (11)	30 (2)	196 (13)	60 (5)
February 2015	1,387	118 (9)	36 (3)	154 (11)	48 (4)
March 2015	1,520	147 (10)	44 (3)	191 (13)	50 (4)
Total	18,493	2,458 (13)	1,037 (6)	3,495 (19)	714 (5)

Note: All appointments booked by appointment letter are included (not appointments booked by telephone)

Users can call or e-mail to give notice of nonattendance or to have their appointment rescheduled. The secretary is open for telephone calls between 0745 and 1430 on weekdays and can be contacted via e-mail 24 hours a day/7 days a week. A short message service (SMS) reminder system is used to prevent nonattendance; however, this can only be used for follow-up appointments, as the user’s phone number is not available until an initial visit has been attended or if the user has attended the clinic before. Based on historical data, we expect that 20–30 % of scheduled users will continue receiving SMS reminders.

### Participants

Consecutive users scheduled for diagnostics, treatment, surgery, or follow-up in the outpatient clinic by appointment letter sent between 1 May 2015 and 1 December 2015 are included. As a user may have more than one appointment, only the first appointment during this period was considered. Users who were issued more than one appointment letter before the first appointment date were excluded. Appointments booked without an appointment letter (e.g., via telephone or face-to-face) are also excluded from the study. Appointments for physiotherapy or occupational therapy, which are typically group-based and scheduled without appointment letters as well as appointments for users residing in Greenland (requiring an overseas flight) are similarly excluded.

### Randomization and consent

User-level 1:1 randomization is conducted according to the time of booking using the electronic patient booking system (BookPlan via MidtEPJ). The booking system automatically generates a random number and assigns the appointment to one of the randomization arms. These data are kept from the secretary and other healthcare professionals unless the user informs them (e.g., in the case of questions or complaints). The researchers will be fully blind throughout the trial and data analysis. The study was considered a quality-improvement project, and informed consent from users was not required by The Ministry of Health and Prevention (See Ethics and data protection). However, users were informed of the risk of receiving a fine on nonattendance at this outpatient clinic via the media and via posters and booklets at the hospital. Furthermore, in Denmark, users have free hospital choice.

### Sample size

Based on department statistics, a nonattendance rate of 4.8 % and a volume of 6,500 eligible appointments during a time window of 7 months are assumed. This time window was adopted to balance of arguments concerning effect size and stability in the study, which are exerted for restructuring and cost cutting after the 7 months. A minimum reduction of 1.58 % (from 4.8 % to 3.22 %) was found using a chi-squared test and a 90 % power level. The randomization ratio is 1:1, which indicates that 3,250 users are required in each arm. The power to detect similar impacts on the secondary outcome parameters of cancellations will be higher due to higher baseline proportions. The power to detect the impact on sociodemographics will depend on the scaling and the relevant level of relative risk reduction [19].

### Comparators

All appointments will be made in accordance with the usual practice except for two aspects. First, the appointment letter will inform the user (for both the intervention and control arms) about the trial. The letter will explain that the investigation has been initiated by the Danish Regions and approved by the Ministry of Health and that the patient’s appointment has been randomly assigned to be subject to a fine in the case of nonattendance without notice by the patient if a letter about the fine system is attached to the appointment letter. Second, for appointments randomized to the intervention group only, a letter specifying the conditions for the fine assessment is attached to the appointment letter. This letter clearly indicates that a fine will be issued in the case of nonattendance if the patient does not give notice prior to the time of the appointment. The patients are also informed that the fine amounts to DKK 250 and that it will be issued by a central office under the local government administration (Corporate Finance, Central Denmark Region), which is open for e-mails and telephone calls during normal office hours if the patient has any questions or complaints.

The secretary registers nonattended appointments in the clinic daily. If a user shows up late, the health professionals will usually attempt to see the user anyways. A list of nonattended appointments will be automatically sent once a week to the central office administering the fine system. Similarly, this office will receive the list of included appointments once a week to handle questions and complaints. Fines will be issued once a week, and recipients are given 4 weeks before the fine is due. In the case of nonpayment, users will receive two reminders; thereafter, the collection of the fine will be handed over to the tax authority (SKAT), which will collect the fine and related expenses.

### Measures of effectiveness

The primary outcome measure is the proportion of appointments that are attended. The secondary outcomes, which are covariates, include the proportions of sociodemographics (age, sex, income, education, distance to hospital, travel time, and waiting times) and health-problem characteristics (anatomy, type of appointment and treatment costs). All parameters except for sociodemographics are automatically extracted from the electronic patient journal system into a trial registry and administratively registered in electronic trial registries. Data on sociodemographics are extracted from various, individual user-level, national registries administered by Statistics Denmark.

### Measures of costs

The opportunity costs are considered from a Danish healthcare perspective and include the intervention cost as well as the productivity costs related to nonattendance. Microcosting of the intervention will be used to estimate the costs of a routine-based system that manages the issuing of fines, questions, complaints, reminders, and the handover of claims to the tax authorities in the case of nonpayment after reminders. The cost determination will be based on detailed time registrations made by the central office undertaking the administration of fines. Time registrations will be integrated in the administrative system at the appointment level and will be valued using average gross staff salaries. The cost of utensils will be added to the standard overhead rate for the administration.

The cost determination of productivity loss due to nonattendance will be estimated by the production value of nonattended appointments if they had been attended. As a worst-case estimate, implying no flexibility of production, the full tariffs of the Diagnosis-Related-Grouping system will be used to value productivity loss. As a best-case estimate, implying that the production is almost fully flexible and will simply take the next user in the case of nonattendance, only a proportion of the production value will be used. The relevant levels of the worst-case and best-case scenarios will be informed from qualitative and observational studies that are conducted in another test department, which is fully exposed to the fine protocol at the departmental level.

It should be noted that revenue from fines is a transfer of money between taxpayers who do not attend scheduled appointments and all other taxpayers (and is not an economic opportunity cost). However, the revenue net of the costs for administrating the fine system is considered for budget impact analysis.

### Analysis of effectiveness

The principle of intention-to-treat will be used for analysis, and each of the outcome parameters will be compared between the intervention and the control groups. A simple test of proportions will be used to test the primary hypothesis. The secondary hypotheses will be tested in two probit regressions: one for user characteristics and one for appointment type characteristics. Both models will have attendance as the dependent variable and a randomization group dummy and user/appointment type characteristics as covariates along with interaction terms for the randomization group dummy and the covariates. The interpretation of the interaction terms will then be the effect modification of the covariates, which is the same as a differentiated effect of fines on different users/types of appointments. A significance level of 0.05 will be used for all analyses.

### Analysis of cost-effectiveness and budget impact

The primary outcome measure will be adopted from the effectiveness analysis (proportion of nonattendance), and the cost parameter will include the net cost of the intervention on the consequences to productivity. The incremental cost effectiveness ratio for the healthcare sector will be analyzed to illustrate the impact of the fines on technical efficiency [20]. The main result will thus be the extra cost of preventing an extra user from not attending, which will be reported as a mean value with 95 % confidence intervals, given that both nominator and denominator are positive; otherwise, the net benefit framework will be adopted to accommodate negative components of the ratio [21, 22]. A sensitivity analysis will be conducted to assess the robustness of the results with respect to methodological choices. The cost-effectiveness analysis will be supplemented with a simple budget impact analysis that considers the revenue from fines less the collection costs [23].

Missing data is not relevant when using register-based data.

The data will be handled in accordance with the Data Protection Act, and the establishment of a research register was approved by the Legal Office at the Central Denmark Region (journal no. 1-16-02-288-15), which has been delegated the authority to handle local research projects by the national Data Protection Agency. Only the researchers authoring this protocol will have access to the data.

## Discussion

The introduction of fines for nonattendance in Danish outpatient clinics has been debated for more than a decade, and stakeholders such as the Danish Regions, Danish Patients, and the Danish Medical Association have been involved in the debate. Although the statutory foundation was found to be present in 2004, no decision was made to conduct the trial until 2013 [14].

The ethics of selecting a particular group of users for nonvolunteer participation in this trial is atypical in comparison with, e.g., clinical experiments where participants are normally only included on giving informed consent. On the other hand, the process of selection reflects the typical rationale for an experimental test in that the effect is examined before eventual implementation to the entire sector. An alternative would be to test the effect of fines on the entire healthcare user population and let users decide whether they will agree to participate in the trial. This was not considered for two main reasons. First, it would require an unrealistic amount of resources to set the intervention up for all healthcare sector providers, and second, we would expect selection into the trial that would introduce severe challenges in terms of external validity. The trial design decisions can thus be said to reflect the ethical principle of utilitarianism [24].

The organization, Danish Patients, was invited to participate as a reference group for the project, which the organization declined. However, the organization agreed to be available for comments and sharing viewpoints on the project.

The main argument in favor of fines has been that public resources should be utilized efficiently. Proponents argue that nonattendance leads to excess labor costs as well as unused facilities and equipment, which will again lead to longer waiting times and/or a more stressful work environment for the staff. Opponents’ main argument is that the use of fines could violate the fundamental value of the Danish healthcare system of free and equal access, and that it provides a threat to the patient-health professional relationship, which should be independent of financial interests [14]. Nevertheless, a number of general population surveys as well as staff expressions in the general media reflect a positive attitude toward the introduction of fines, and similar fines are already used elsewhere in the public sector [14].

According to neoclassical economic theory, users will attend an appointment if the personal benefits exceed the expected costs associated with committing an offense [11]. Monetary fines thus influence people’s behavior, and, consequently, the proportion of non-attendees will decrease when fines are introduced. This also means that the higher the fine is, the more significant the behavioral change will be. The level of fines in this study is a politically set value, although it should be noted that, ideally, the dose–response relationship between fine level and nonattendance should have been assessed empirically. The level chosen is a reflection of what is already allowed by the collaborative agreement between the Danish Regions and the Danish Medical Association for privately practicing specialists [15].

Also according to neoclassical economic theory, the level of a fine will have different effect across users with different income. The level of DKK 250 corresponds to, e.g., a regional return ticket for the train or ten newspapers. The Ministry of Health decided that this trial should adopt this level because fines of DKK 250 are already used elsewhere in the public sector. Estimation of intervention costs as well as the value of improved productivity (cost saving) is a part of the planned cost effectiveness. The revenue from fines is expected to sum to a limited amount, as the maximum effect potential (until nonattendance) of less than 5 % multiplied by the DKK 250, is quickly outweighed by intervention costs. The revenue from fines will be quantified as an economic redistribution from those who do not attend to those who attend, and the revenue will be discussed relative to the implications for equity and ethics.

Because the orthopedic clinic is simultaneously exposed to fines and to no fines, we cannot evaluate “overhead” provider-level consequences such as improved productivity. In principle, we could alternatively have randomized a number of departments to fine-based practice versus no-fine-based practice, but this would require an unrealistic amount of trial resources and involve hundreds of thousands of patients. Given this reality, we have chosen to focus on the first-line consequence of fines, namely user response to this risk of financial penalty. However, longer-term consequences of, e.g., productivity or even health effects for users, could be linked to attendance. An important limitation of this study is that we only assess the user-level effects.

The provider-level effects are, however, being investigated in a parallel, nonrandomized intervention study at a diagnostic center of a different hospital in another city (Diagnostic Centre at Silkeborg Regional Hospital), which is exposed to fines for a 12-month period from December 2015. While that study potentially suffers bias from the nonrandomized design, the aim is to inform relevant the provider-level effects such as production volume, staffing, staff sick leave, ability to conform with political targets for time to diagnosis, and overtime work, which can be interpreted as provider-level effects that are linked to users attending appointments.

The generalizability of the trial is restricted by the choice of the orthopedic setting. However, given the scale of the trial, the availability of detailed user characteristics and because the orthopedic department serves quite diverse users, we will be able to generate weights for the effect of nonattendance that can be used to predict the effect for different user populations. What cannot be assessed analytically, however, are the alternative contextual characteristics such as infrastructure, culture, and hospital characteristics. In a previous survey, nonattendance rates across eight different specialties were found to differ at the provider level, whereas the overall level across regions was found to be similar [14]. In a recent observational study at a gastroenterology outpatient clinic, the level of nonattendance was found to be 6.1 % after the introduction of telephone reminders [25], which is above the historical level in the current setting.

The perspective of the trial includes national policymaking about healthcare management. The findings of the trial are expected to inform political decisions about the introduction of fines as a general premise in the context of Danish hospitals.

### Trial status

The trial began accepting users on 1 May 2015. The design and preparation of the randomization and data protection procedures, the agreements of collaboration with clinical department, and all ethical approvals have all been settled. The trial closed on 1 December 2015.

## Abbreviations

CONSORT, Consolidated Standards of Reporting Trials; DKK, Danish kroner; RCT, randomized controlled trial; SMS, short message service
